# Effects of Climatic Change on Phylogeography and Ecological Niche of the Endemic Herb *Elymus breviaristatus* on the Qinghai-Tibet Plateau

**DOI:** 10.3390/plants12183326

**Published:** 2023-09-20

**Authors:** Jin Li, Changbing Zhang, Tserang Donko Mipam, Qingping Zhou, Shiyong Chen

**Affiliations:** 1 Sichuan Zoige Alpine Wetland Ecosystem National Observation and Research Station, Southwest Minzu University, Chengdu 610041, China; 2 Sichuan Academy of Grassland Science, Chengdu 610041, China; 3 College of Animal and Veterinary Sciences, Southwest Minzu University, Chengdu 610041, China

**Keywords:** *Elymus breviaristatus*, chloroplast DNA, phylogeography, Qinghai-Tibet Plateau, genetic barrier, ecological niche

## Abstract

Past climatic and topographic variations have created strong biogeographic barriers for alpine species and are key drivers of the distribution of genetic variation and population dynamics of species on the Qinghai-Tibet Plateau (QTP). Therefore, to better conserve and use germplasm resources, it is crucial to understand the distribution and differentiation of genetic variation within species. *Elymus breviaristatus*, an ecologically important rare grass species with strong resistance, is restricted to a limited area of the QTP. In this study, we investigated the phylogeography of *E. breviaristatus* using five chloroplast genes and spacer regions in natural populations distributed along the eastern QTP. We identified a total of 25 haplotypes among 216 individuals from 18 *E. breviaristatus* populations, which were further classified into four haplogroups based on geographical distribution and haplotype network analysis. Notably, we did not observe any signs of population expansion. High genetic diversity was exhibited at both species and population levels, with precipitation being the main limiting factor for population genetic diversity levels. Higher genetic diversity was exhibited by populations located near the Mekong–Salween Divide genetic barrier, suggesting that they may have served as a glacial refuge. The significant pattern of genetic differentiation by environmental isolation highlights the influence of heterogeneous environments on the genetic structure of *E. breviaristatus* populations. Additionally, the results of ecological niche models indicated that the geographic distribution of *E. breviaristatus* populations has decreased rapidly since the Last Glacial Maximum but is not threatened by future global warming.

## 1. Introduction

The spatial distribution of genetic variations in populations has been shaped by population expansion and contraction triggered by geological events or climatic fluctuation [1,2]. Specifically, past climate fluctuations, particularly since the Quaternary period, have caused repeated shocks of cold and warm climates due to alternating glacial and interglacial periods [3]. In response to rapid climate change, natural populations may migrate to more favorable locations, adapt locally to novel environments, or face extinction [4]. The geographic distribution and genetic structure of the majority of species in the northern hemisphere have been profoundly influenced by the abovementioned phenomena [1]. In addition, habitat fragmentation and the emergence of exotic distributions of different species or intraspecific genetic changes may have been caused by repeated environmental changes [5]. Phylogeography, which uses molecular data to reconstruct historical biogeography, is a valuable approach to understanding these changes [6]. Previous studies have analyzed genetic structures among extant species to facilitate the understanding of historical changes in species distribution and speciation [7]. Additionally, insights into a species’ geographic distribution can be obtained through ecological niche modeling (ENM), and it can also predict changes in distribution using species occurrence data and environmental conditions [8]. When combined with phylogeography, ENM can assess the effects of climate change on species distribution patterns and reveal the processes that shape the current distribution [9].

The Qinghai-Tibet Plateau (QTP) is located in the southwestern region of China and is a geographically remarkable area that is the highest and largest plateau on the planet, with an average elevation of over 4000 m [10]. During the uplift between the Miocene and Quaternary periods, the QTP underwent significant changes in its geographical and climatic features, resulting in its recognition as one of the most important biodiversity hotspots worldwide [11,12]. Moreover, the QTP is critical for biodiversity conservation and research because it provides essential ecosystem services [13]. Its exceptional plant diversity and unique ecological characteristics are attributed to its complex topography, heterogeneous geological substrate, and extreme climate, among other factors [14]. However, climate warming is a significant threat to global biodiversity, particularly for endemic species in alpine regions that are highly sensitive to anthropogenic global warming [15,16]. The pattern of alpine species migrating toward higher altitudes in response to climate change has been documented in many studies [15,17]. In their recent study, Liang et al. reported that while many alpine plants in the QTP–Hengduan Mountains may not be at a high risk of extinction due to habitat contraction induced by climate warming, certain species at extremely high altitudes exhibit significant range contractions [18]. This supports the “mountain-top” extinction and upslope contraction hypothesis [19], indicating that conservation measures are necessary for species inhabiting very high altitudes or having narrow distributions. While this evidence suggests that global climate change is not a serious threat to a range of alpine plants, its influence on genetic diversity has been largely overlooked, probably due to the difficulty in assessing it [20]. Genetic diversity is the primary basis for the evolution of natural selection, and it plays a pivotal role in determining how species respond to the challenges of future climate change [21]. Hence, investigating the spatial patterns of genetic variation within species can help identify conservation priorities for specific populations and evaluate the adaptive and migratory potential of populations in response to climate change.

Chloroplast DNA (cpDNA) is a valuable tool for investigating phylogeography, primarily due to its maternal inheritance in most angiosperms and its limited genetic recombination [22]. These characteristics enable straightforward tracking of geographical patterns of variation, making cpDNA an advantageous molecular marker for understanding plant migration and history, as well as identifying populations that may be at risk of extinction. Using molecular data for phylogeography, researchers can effectively analyze genetic variation and identify potential barriers to differentiation in plant populations [23,24]. Several discernable geographical barriers in the QTP region have been identified in previous studies, such as the Mekong–Salween Divide (MSD) and the Tsangpo–Brahmaputra Grand Canyon [24,25,26]. As a result, the QTP is a crucial area for exploring phylogeography. A better understanding of the function of these barriers can provide valuable insights into the evolutionary history of plant diversity in the floristic regions of the QTP [25].

In this study, we used both environmental data and phylogeography to investigate the factors affecting genetic diversity in *Elymus breviaristatus* (Keng) Keng f. (Poaceae: Triticeae), a self-pollinating grass species endemic to the QTP region that is at risk of extinction due to habitat fragmentation [27]. This species belongs to the genus *Elymus*, characterized by a complex taxonomic history shaped primarily by hybridization processes [28]. Specifically, *E. breviaristatus*, the focus of our study, is a hexaploid species with a genome comprising St, H, and Y genomes, exhibiting rich genetic diversity and a high frequency of gene exchange. Given its rarity, short life cycle, and sensitivity to environmental changes, *E. breviaristatus* represents an excellent model for studying the evolution and genetic variation in alpine plants [29]. Furthermore, the richness of resistance genes in herbaceous plants has been demonstrated in numerous studies [30], emphasizing the importance of conserving germplasm resources for plant breeding [31]. To explore the evolutionary history of *E. breviaristatus*, we employed a combination of cpDNA analysis and ENM, as well as the geological and climatic changes that may have influenced its distribution shift and genetic diversity. The findings of this study can contribute to the identification of factors contributing to species’ endangerment, germplasm resource assessment, and the development of effective conservation strategies.

## 2. Results

### 2.1. Haplotype Distribution and Phylogenetic Relationship

**Figure 1:**
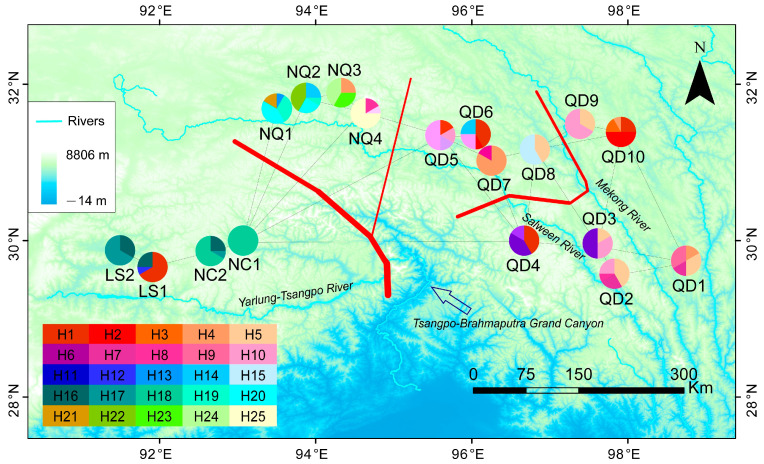
Geographical location of 18 populations of *Elymus breviaristatus* and distribution of 25 cpDNA haplotypes. The red lines in the map represent the genetic disorders revealed by using BARRIER 2.2 software.

The total length of the aligned sequences for the five chloroplast fragments was 5113 bp. The lengths of the *trn*H-*psb*A, *trn*L-*trn*F, *mat*K, *rbc*L, and *trn*Y_GUA-*trn*D_GUC regions were 667, 1052, 1538, 1432, and 384 bp, respectively. A total of 36 variable sites were detected, comprising one singleton variable site and 35 parsimony informative sites. As cpDNA regions are uniparentally inherited markers, we used these five chloroplast fragments in our subsequent population genetics analysis. A total of 25 haplotypes (H1–H25) were identified among 216 individuals of *E. breviaristatus* sampled from 18 populations (Figure 1 and Table 1). Haplotypes H1 and H10 were the most common, each with a frequency of 9.72% (21 individuals), and were mainly distributed in Qamdo, with occurrences also in Lhasa and Nagqu. Unique haplotypes were detected in three regions: Qamdo (H2, H3, H5–H7, H9, H11, and H15), Nagqu (H19–H25), and Nyingchi (H18). No unique haplotypes were found in Lhasa.

**Figure 2:**
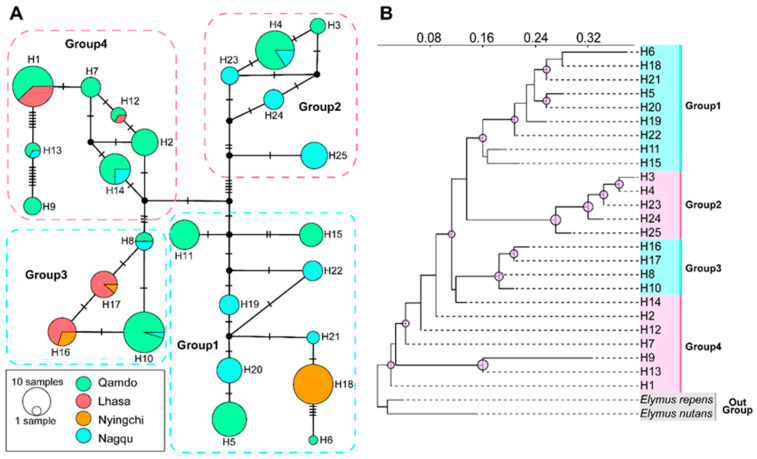
Haplotype structure of cpDNA sequences. (**A**) Network of cpDNA haplotypes of *Elymus breviaristatus*. The color combinations of each circle (H1–H25) represent the distribution in different regions, and the size of the circles is proportional to the frequency of each haplotype in the entire sample of the species. (**B**) Maximum likelihood tree for cpDNA haplotypes, where the circles above the branches indicate bootstrap values greater than 0.5.

Based on geographic distribution, haplotype network (Figure 2A), and the ML tree (Figure 2B), the 25 haplotypes were divided into four distinct haplogroups. Group 1 contained nine widespread haplotypes that did not occur in Lhasa. Group 2 contained five haplotypes distributed only in the Qamdo and Nagqu populations. Group 3 comprised four haplotypes forming a closed ring: H8 and H10 occurred in both Qamdo and Nagqu, while H16 and H17 occurred in both Lhasa and Nyingchi. Group 4 included two unique Qamdo haplotypes (H7 and H9), as well as common haplotypes shared between Qamdo–Lhasa and Qamdo–Nagqu.

### 2.2. Population Genetic Diversity and Dynamics

**Figure 3:**
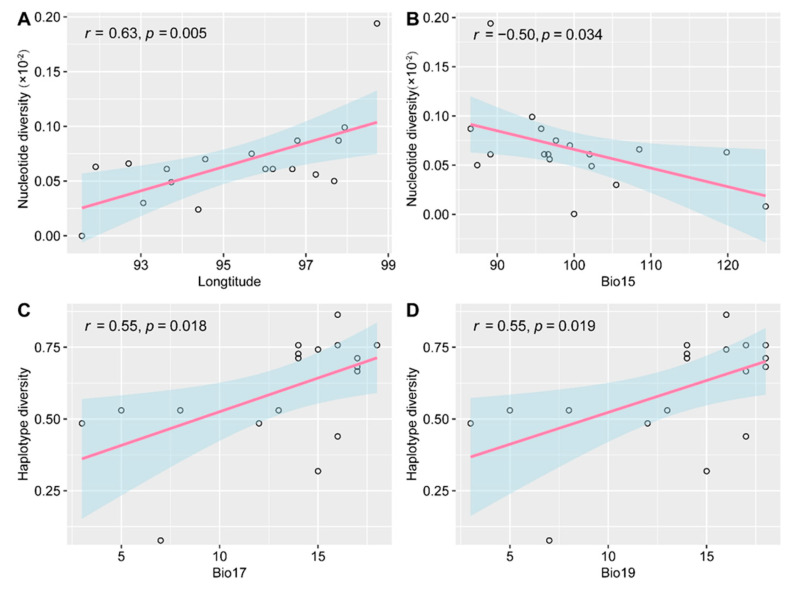
Pearson correlation coefficients for levels of genetic diversity with geographic and environmental conditions. (**A**) Longitude and nucleotide diversity, (**B**) Bio15 (precipitation seasonality) and nucleotide diversity, (**C**) Bio17 (precipitation of driest quarter) and haplotype diversity, and (**D**) Bio19 (precipitation of coldest quarter) with haplotype diversity. The blue shaded represent 95% confidence interval.

The genetic diversity of the 18 populations is shown in Table 2. At the species level, *E. breviaristatus* exhibited high genetic diversity with a haplotype diversity (*Hd*) of 0.825 and a nucleotide diversity (π × 10^−3^) of 1.14. At the population level, *Hd* ranged from 0 to 0.818 and π × 10^−3^ varied from 0 to 2.3. Population QD1 had the highest haplotype and nucleotide diversity, with six haplotypes, while population NC1 had only one haplotype and no nucleotide diversity. The effects of geographical and climatic conditions on haplotype and nucleotide diversity were assessed using Pearson correlation (Figure 3). The nucleotide diversity increased with longitude (*r* = 0.63, *p* = 0.005) but decreased with Bio15 (precipitation seasonality, *r* = −0.5, *p* = 0.034). Haplotype diversity was significantly affected by Bio17 (precipitation of the driest quarter, *r* = 0.55, *p* = 0.018) and Bio19 (precipitation of the coldest quarter, *r* = 0.55, *p* = 0.019). Additionally, the AMOVA indicated considerable genetic variations among and within populations (48.9% and 51.1%, respectively; Table 3).

Mismatch distribution was used to observe the difference in the position of paired nucleotides; the results of Tajima’s *D* and Fu’s *F*s tests of neutrality were not significant among all individuals or within each of the 18 populations (Table 2 and Figure S1). In addition, no significant Tajima’s *D* and Fu’s *F*s values were detected in the four haplotype groups we identified (Table S4), and none of the haplogroups showed a unimodal curve (Figure S1), suggesting no signals of population expansion in the analysis.

### 2.3. Genetic Differentiation Barriers and Isolation Models

BARRIER 2.2 was used to determine genetic barriers among populations (the red line in Figure 1). An obvious genetic barrier was detected between the populations from Nyingchi and Lhasa and the populations from other areas, which was geographically coincident with the Nyenchen Tanglha Mountains and Tsangpo–Brahmaputra Grand Canyon combination. The second major barrier was detected in the Qamdo area, isolating four populations (QD5–QD8) from other populations from Qamdo; coincidentally, these four populations were all distributed between the Salween River and the Mekong River.

**Figure 4:**
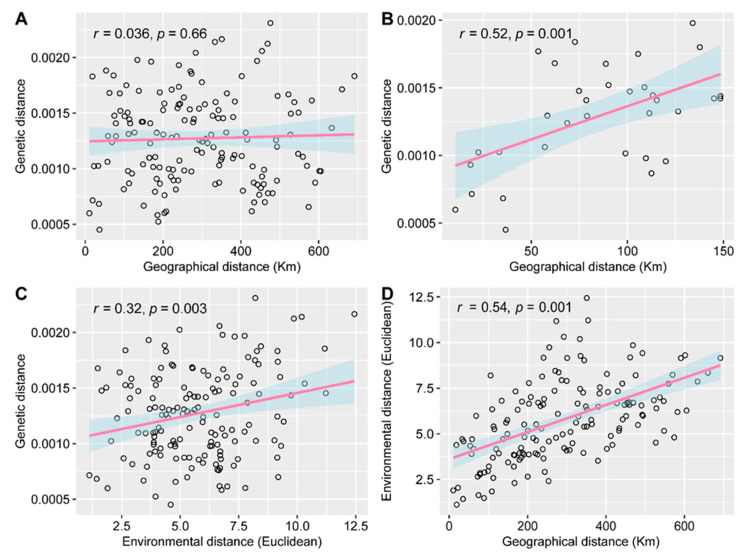
Linear regression lines showing correlations among genetic, geographical, and environmental distances. (**A**) Genetic and geographical distances, (**B**) genetic and geographical distances when the geographical distance is less than 150 km, (**C**) genetic and environmental distances, and (**D**) geographical and environmental distances. The blue shaded represent 95% confidence interval.

These genetic barriers suggest that *E. breviaristatus* populations correspond to the IBD pattern to some extent. However, our Mantel test found no significant correlation between population genetic distance and geographic distance until we controlled the geographic range within 150 km (Figure 4A,B). In addition, a significant correlation between population genetic distance and environmental distance suggested that genetic differentiation could be explained by IBE (Figure 4C), indicating that the heterogeneous environment affected the genetic structure of *E. breviaristatus* populations. Nevertheless, the significant autocorrelation between geographical and environmental distances implies that the greater the geographical distance, the greater the difference in environmental characteristics (Figure 4D).

### 2.4. Species Distribution Change

In this study, we used MaxEnt to model the potential distribution of the species and evaluated the predictive capacity of the model using AUC values. The high AUC values (≥0.927) indicated that the MaxEnt model had a good predictive capacity. Through an analysis of the contribution rates of seven environmental variables to the distribution of *E. breviaristatus* (as delineated in Table S3) and the integration of outcomes derived from the Jackknife method, it becomes evident that annual precipitation (bio12) emerges as the primary determinant impact with a substantial contribution rate of 42.06%. Subsequently, altitude assumes significance, accounting for 29.61% of the influence, while the max temperature of the warmest month (bio5) follows with a contribution rate of 10.09%, signifying their significant roles in shaping the suitable distribution habitats of *E. breviaristatus*.

**Figure 5:**
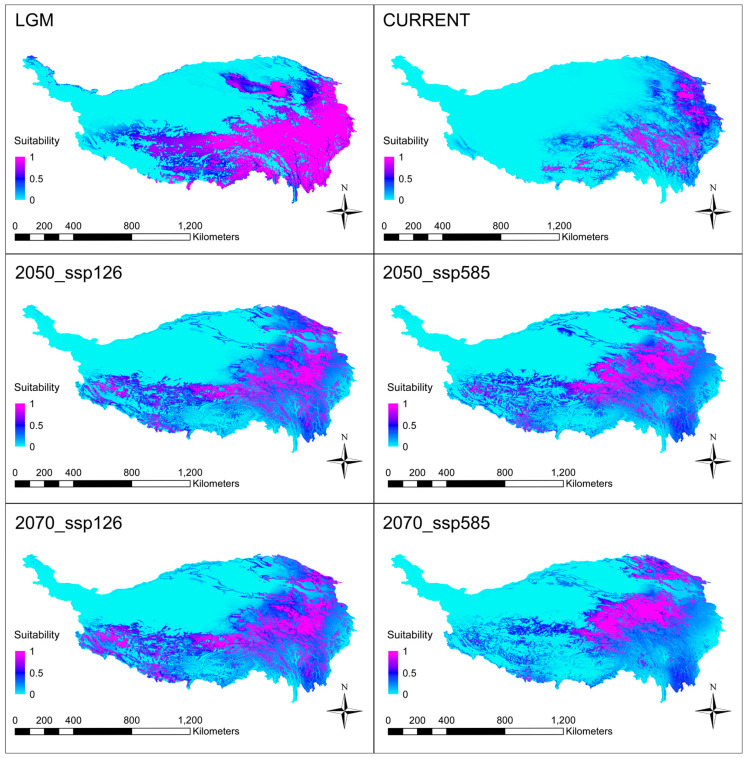
Geographic distribution pattern obtained for *Elymus breviaristatus* using MaxEnt. LGM, last glacial maximum, ~21,000 years before present; CURRENT, 1950–2000.

Projecting the model into geographical space predicted the potential distribution of the species from the LGM to the present. The species’ potentially suitable habitats have undergone a rapid contraction since the LGM. The current potential distribution areas included the known distribution of species in the southeast of the QTP, involving some adjacent areas of Tibet, Qinghai, Gansu, Sichuan, and Yunnan. In addition, the predicted future distribution range of the species showed different ranges of expansion under different scenario models. Of note, the distribution area will migrate to the northwest in the future (Figure 5).

## 3. Discussion

### 3.1. Spatial Distribution of Genetic Diversity

The limited distribution of a species can lead to reduced gene flow and increased genetic drift, which can result in lower genetic diversity. Since *E. breviaristatus* is endemic to the QTP and has a narrow distribution, it was initially expected to have lower genetic diversity compared with other *Elymus* species. However, the present study revealed a high *Hd* (0.825) and π (0.00114), which was comparable to the genetic diversity of the widespread species of *Elymus sibiricus* in the QTP region detected based on cpDNA (*Hd* = 0.834, π = 0.00108) and even higher than the genetic diversity of *E. sibiricus* in Xinjiang and northern China (*Hd* = 0.527 and 0.543, π = 0.00042 and 0.00053, respectively) [32]. The high level of genetic diversity observed in *E. breviaristatus* could be due in part to the large number of samples in the study. However, this is also consistent with previous studies that have found high levels of genetic diversity in plant species on the QTP [33,34]. The unique environment and history of the plateau, including its high altitude, extreme climate, and geological and ecological complexity, are thought to have contributed to the high levels of genetic diversity in many plant species found there [34]. Furthermore, differences in ploidy levels between species could contribute to this phenomenon [35]. The hexaploid *E. breviaristatus* may have accumulated a higher genetic variation in its chloroplast genome during its lengthy evolution and polyploidization history than the tetraploid *E. sibiricus*.

Considerable variation in genetic diversity was observed among the populations studied, with the population from the eastern part of the study area QD1 exhibiting high levels of haplotype and nucleotide diversity, while NC1 from the western part showed no variation. Although the correlation between genetic variation and geographical features, such as altitude and latitude, has been highlighted in previous studies [36,37], our investigation reveals a different pattern. Specifically, we found that only longitude had a statistically significant influence on nucleotide diversity, with a gradient of decreasing diversity from east to west, consistent with our earlier study of these populations using microsatellites [38]. The sampling strategy used in this study might have not fully accounted for the effects of latitude or altitude on genetic diversity, or confounding factors such as gene flow and local adaptation were not adequately considered. Nonetheless, similar trends were observed by Ye et al. [39], suggesting that climatic factors at different longitudes may limit the level of genetic diversity in populations. Our analysis of the correlation between climate factors and genetic diversity revealed that stable seasonality of precipitation and elevated precipitation during extremely dry or cold seasons were positively associated with population genetic diversity. These results are consistent with previous findings [38]. However, it is important to note that future projections predict that the temporal distribution of precipitation will become more heterogeneous due to global warming, resulting in stronger fluctuations during dry and wet periods [40]. This suggests that the genetic diversity of *E. breviaristatus* may decrease in the future due to increased precipitation instability.

### 3.2. Genetic Differentiation Caused by Barriers and Environmental Heterogeneity

Gene exchange plays a critical role in plant evolution and population structure [41]. Our AMOVA revealed that almost half of the genetic variation was observed between populations, indicating restricted gene flow between some populations. In addition, the Nyenchen Tanglha Mountains and Tsangpo–Brahmaputra Grand Canyon combination was identified as one of the most significant barriers through our barrier analysis, which has been repeatedly emphasized in previous studies, not only in this species but also in other plant species, such as *Koenigia forrestii* and *Roscoea* spp. [23,24]. Another barrier was found between the Mekong and Salween rivers. Interestingly, the Ward Line–MSD has been identified as an important flora boundary for the QTP, as evidenced by other plant species, such as *Marmoritis complanatum* and *Sinopodophyllum hexandrum* [25,42]. Our findings confirm the influence of the MSD as a biogeographic barrier on the population structure of the endemic plant *E. breviaristatus* in the QTP region and shed light on why the eastern populations exhibit a higher genetic diversity. This could be attributed to their proximity to the MSD, which may serve as a hybridization zone for different species or populations and represent a transition zone between distinct environmental conditions [43].

The presence of geographic barriers can limit gene flow between populations, leading to the rapid accumulation of genetic differentiation, while also constraining the effective distance of gene transmission, ultimately affecting the differentiation of IBD patterns [44]. However, our investigation revealed no significant IBD patterns in all populations until we limited the geographic range to 150 km, a phenomenon also observed in the previous results of Chen et al. [45] for the closely related species *E. nutans*. Nonetheless, IBE patterns were detected in all populations. By combining the autocorrelation between environmental distance and geographic distance, we infer that the genetic differentiation pattern of *E. breviaristatus* is dominated by IBE, which is consistent with our previous microsatellite-based findings [38]. The pattern of IBD on a small scale may arise due to the emergence of geographic barriers or reflect differential adaptation to heterogeneous environments, which further leads to pseudo-correlation for IBD [46]. In conclusion, we propose that the differential adaptations of *E. breviaristatus* populations to heterogeneous environments are a more plausible explanation for the observed differential selection than the neutral process of IBD.

### 3.3. Population Dynamics and Ecological Niche

Although neutrality tests and mismatch analyses did not indicate a significant influence of selective events on all of our populations and haplogroups, it is important to note that Tajima’s *D* and Fu’s *F*s values were higher than 0 for most populations in the neutrality tests. This suggests that some degree of population contraction may have occurred [47,48], albeit not significant. When considering the species distribution model results, it is evident that the distribution range of *E. breviaristatus* has experienced a significant decline since the LGM, with a contraction to higher elevations in the mountains. This decline in distribution range may have been caused by past selection pressure or the neutral drift experienced by these populations [49].

In contrast to previous studies that suggested that montane species face range contraction or extinction as they shift upward in response to global warming [50], our study found that the distribution range of *E. breviaristatus* will increase and shift to higher elevations in the central QTP, and this trend will be even more pronounced with increasing warming. Previous research by Elsen and Tingley [51] predicted that the range of montane species may increase in the future. Furthermore, a recent simulation study of 151 species in the Hengduan Mountains also showed an increase in range size under a warming climate, and the distribution range of species was likely to shift to higher elevations northward and westward [18]. Our findings support these views. Based on our simulations, it appears that the alpine herb *E. breviaristatus* is not at a high risk of range contraction or extinction due to climate warming in the future.

### 3.4. Implications for Conservation and Management

The highly endemic montane plants are known to be particularly vulnerable [50], and despite our ENM results indicating that the distribution range of *E. breviaristatus* is not currently affected by climate change, habitat fragmentation resulting from anthropogenic disturbances remains a major concern for endangered species [52]. The longstanding pressures of overgrazing and artificial development on terrestrial ecosystems in the QTP region pose significant threats to the species [53]. Additionally, we cannot overlook the genetic erosion of natural populations caused by sandy land management practices and the establishment of artificial grasslands using *E. breviaristatus* [38].

Therefore, our recommendation is to prioritize the conservation of natural populations that are distributed near the MSD due to their high haplotype diversity and potential as glacial refuges. These populations possess abundant genetic diversity, which provides them with increased adaptability and resistance to selection pressures [54]. Additionally, we suggest collecting seeds for the establishment of germplasm nurseries for allopatric conservation, with a focus on populations that exhibit a high frequency of private haplotypes, such as those found in Nagqu. These unique genetic components can be used to maintain the genetic diversity and adaptive capacity of the species and mitigate the risk of extinction. Furthermore, given the presence of numerous closely related, widespread species of *E. breviaristatus* in the QTP, including *E. nutans* and *E. sibiricus*, we recommend incorporating hybridization between different populations or species in the conservation process. This promotes the extensive exchange of genetic information and further enhances the adaptive capacity of the species [55].

## 4. Materials and Methods

### 4.1. Population Sampling and DNA Extraction

A total of 216 silica-dried leaf samples were collected from 18 populations of *E. breviaristatus* individuals across the QTP. The seeds of these specimens were planted at the experimental field of the Sichuan Academy of Grassland Science, Hongyuan, China. These comprised 10 from Qamdo (QD1–QD10), 4 from Nagqu (NQ1–NQ4), 2 from Nyingchi (NC1 and NC2), and 2 from Lhasa (LS1 and LS2). Within each population, 12 individuals were sampled at least 5 m apart. Table S1 provides detailed information about the collection sites, geographical coordinates, and elevations for each source population. The DP350 Plant DNA Kit (Tiangen Biotechnology, Beijing, China) was used to extract genomic DNA following the manufacturer’s protocol. A NanoDrop-Lite instrument (Thermo Scientific, Waltham, MA, USA) and 1% agarose gel were used to determine the quality and quantity of the DNA samples, respectively.

### 4.2. Chloroplast DNA Sequencing

Our pilot screen of 16 individuals from different populations of *E. breviaristatus* using 10 cpDNA regions identified 5 markers (*trn*H-*psb*A, *trn*L-F, *mat*K, *rbc*L, and *trn*Y_GUA-*trn*D_GUC; Supplementary Materials, Table S2) with sequence variability, which were then used for the full survey of all 216 individuals. Amplification was performed in a reaction volume of 30 μL, containing 30 ng of genomic DNA, 1.5 μL of each primer, and 15 μL of 2× Es Taq MasterMix (CoWin Biosciences, Beijing, China), using a C1000 Touch Thermal Cycler (BIO-RAD, Foster City, CA, USA). The polymerase chain reaction (PCR) conditions during the entire experiment were as described by Shaw et al. [56] and Tsingke Biotech (Beijing, China) sequenced the products. All DNA sequences were edited and adjusted using DNAMAN to obtain consensus sequences and aligned using the ClustalW algorithm in MEGA 6.0 [57]. PhyloSuite v1.2.3 [58] was used to concatenate the sequences serially, and the resultant concatenated sequences were used for subsequent phylogeographic analysis.

### 4.3. Genetic Diversity and Phylogeographic Analysis

DnaSP version 5.0 [59] was used to calculate haplotype (*H*), haplotype diversity (*Hd*), nucleotide diversity (π), and population genetic distance based on concatenated sequences. A haplotype network was inferred using the median-joining method in PopART version 1.7 [60]. To construct a maximum likelihood (ML) tree with 1000 bootstrap replications, phylogenetic analysis was performed using MEGA 6.0. The Interactive Tree of Life (iTOL v4) online tool [61] was used to edit the resulting trees, with *Elymus nutans* and *Elymus repens* as outgroups. DnaSP version 5.0 was used to calculate Tajima’s *D* [47] and Fu’s *F*s [48] to detect potential population spatial expansion through mismatch distribution analysis and neutrality tests. Arlequin version 3.5 [62] was used to conduct an analysis of molecular variance (AMOVA) to estimate genetic variance within and between populations. Additionally, BARRIER 2.2 was used to identify barriers that reduce gene flow within species distributions based on Monmonier’s maximum difference algorithm [63].

### 4.4. Genetic Differentiation

Genetic drift typically results in greater differentiation among populations at larger geographical distances, assuming no dispersal or selection. This phenomenon is known as isolation by distance [64] (IBD). However, IBD does not account for the influence of environmental factors on gene flow, which is referred to as isolation by environment [65] (IBE). In this study, we used a Mantel test to assess the effects of geographical distance and environmental differences on genetic structure among populations. The geosphere package in R was used to calculate pairwise geographical distances using latitude and longitude data. Nineteen bioclimatic variables obtained from the WorldClim website (https://www.worldclim.org/) were used to compute environmental Euclidean distances. We evaluated IBD and IBE by regressing genetic distance against geographical distance and environmental Euclidean distance, respectively.

### 4.5. Ecological Niche Modeling

To predict the ecological niche distribution of *E. breviaristatus* in the QTP, we used a maximum entropy model (MaxEnt) [66]. In addition to the 18 distribution records obtained in this study, we used 25 additional records from museum specimens and literature sources, for a total of 43 records. We collected 19 bioclimatic variables (as selected in the IBE test) from WorldClim for two periods: the Last Glacial Maximum (LGM; approximately 22,000 years ago) and 1970–2000 (current). These data were used to explore potential habitats and infer past demographic processes from paleoclimate data. We also used two future scenario models (ssp_126 and ssp_585) for two periods (2050 and 2070) to assess changes in habitat suitability. To avoid overfitting or redundancy due to complex variables, we excluded bioclimatic variables with contribution values <5% or correlations >0.8. This resulted in a final set of six climate variables (Bio5, Bio7, Bio12, Bio14, Bio15, and Bio17) and altitude for model building (Table S3). During modeling, we used 75% of the distribution records for training and 25% for testing, with 10 replicates.

## 5. Conclusions

In this study, we present the first phylogeographic analysis of *E. breviaristatus*, revealing that the species has not undergone significant population expansion. Our results indicate that precipitation under extreme climatic conditions is the primary limiting factor for genetic diversity. We observed that populations located near the MSD exhibited higher genetic diversity, suggesting that these regions may have served as refugia. Population differentiation was predominantly determined using the IBE model, with some evidence supporting the IBD model on a smaller scale due to geographical barriers. While our ecological niche analysis provides a more optimistic outlook for the future distribution of this species, we recommend that appropriate conservation measures be implemented to safeguard the population from potential anthropogenic threats.

## Figures and Tables

**Table 1:** Distribution of *Elymus breviaristatus* haplotypes in individuals, populations, and regions.

Haplotypes	Number/Frequency (%) of Individuals	Number/Frequency (%) of Populations	Included Populations	Geographical Distributions
H1	21/9.72	4/22.22	QD10, QD4, QD6, LS1	Qamdo and Lhasa
H2	9/4.17	3/16.67	QD10, QD5, QD6	Qamdo
H3	4/1.85	2/11.11	QD10, QD1	Qamdo
H4	19/8.80	4/22.22	QD10, QD7, QD8, NQ3	Qamdo and Nagqu
H5	15/6.94	4/22.22	QD1, QD2, QD3, QD9	Qamdo
H6	1/3.24	1/5.56	QD1	Qamdo
H7	7/3.24	3/16.67	QD1, QD2, QD7	Qamdo
H8	3/1.39	2/11.11	QD2, NQ4	Qamdo and Nagqu
H9	4/1.85	1/5.56	QD1	Qamdo
H10	21/9.72	6/33.33	QD2, QD3, QD5, QD6, QD9, NQ4	Qamdo and Nagqu
H11	11/5.09	2/11.11	QD3, QD4	Qamdo
H12	3/1.39	2/11.11	QD4, LS1	Qamdo and Lhasa
H13	3/1.39	2/11.11	QD5, NQ1	Qamdo and Nagqu
H14	12/5.56	3/16.67	QD5, QD6, NQ2	Qamdo and Nagqu
H15	7/3.24	1/5.56	QD8	Qamdo
H16	10/4.63	3/16.67	LS1, LS2, NC2	Lhasa and Nyingchi
H17	9/4.17	2/11.11	LS2, NC2	Lhasa and Nyingchi
H18	20/9.26	2/11.11	NC1, NC2	Nyingchi
H19	5/2.31	2/11.11	NQ1, NQ2	Nagqu
H20	8/3.70	2/11.11	NQ1, NQ2	Nagqu
H21	2/0.93	1/5.56	NQ1	Nagqu
H22	5/2.31	1/5.56	NQ2	Nagqu
H23	4/1.85	1/5.56	NQ3	Nagqu
H24	5/2.31	1/5.56	NQ3	Nagqu
H25	9/4.17	1/5.56	NQ4	Nagqu

**Table 2:** Summary of cpDNA variation for 18 populations of *Elymus breviaristatus*.

Population	Sample Size	Number of Haplotypes	*Hd*	π × 10^−3^	Tajima’s *D*		Fu’s *F*_s_	
					*D*	*p*-Value	*F*s	*p*-Value
QD1	12	6	0.818	2.300	0.615	0.731	3.711	0.960
QD2	12	4	0.758	1.040	1.879	0.982	3.863	0.958
QD3	12	3	0.667	0.600	1.203	0.904	3.595	0.940
QD4	12	3	0.682	0.730	2.354	0.998	4.342	0.971
QD5	12	4	0.727	0.910	−0.028	0.523	3.350	0.941
QD6	12	4	0.758	0.730	1.625	0.973	2.636	0.895
QD7	12	2	0.318	0.730	−0.864	0.224	4.326	0.960
QD8	12	2	0.530	0.940	2.441	1.000	8.129	0.998
QD9	12	2	0.485	0.670	1.816	0.980	6.438	0.992
QD10	12	4	0.712	1.190	0.919	0.849	4.387	0.959
LS1	12	3	0.530	0.760	1.162	0.898	4.468	0.969
LS2	12	2	0.485	0.100	1.066	0.904	1.003	0.567
NC1	12	1	0.000	0.000	0.000	1.000	0.000	N.A.
NC2	12	3	0.530	0.800	1.451	0.933	4.684	0.980
NQ1	12	4	0.742	0.730	−1.490	0.081	2.610	0.895
NQ2	12	4	0.758	0.590	1.177	0.905	1.996	0.861
NQ3	12	3	0.712	0.290	1.573	0.951	1.526	0.816
NQ4	12	3	0.439	0.840	0.703	0.796	4.896	0.977
All	216	25	0.825	1.140	0.686	0.713	0.129	0.635

Note: *Hd* = haplotype diversity; π = nucleotide diversity; N.A. = not applicable.

**Table 3:** Analysis of molecular variance (AMOVA) for 18 populations of *Elymus breviaristatus* based on chloroplast DNA sequence data.

Source of Variation	*d.f.*	SS	Variance Components	Percentage of Variation	*p*-Value
Among populations	17	416.963	1.88022	48.9	<0.001
Within populations	198	389	1.96465	51.1	<0.001
Total	215	805.963	3.84486		

Note: *d.f.* = the degree of freedom; SS = sum of squares.

## Data Availability

DNA sequences: deposited in Genbank under accessions OQ772327–OQ773406.

## Supplementary Materials

The following supporting information can be downloaded at: https://www.mdpi.com/article/10.3390/plants12183326/s1, Table S1: Geographic information of 18 *Elymus breviaristatus* populations; Table S2: Screening for qualified cpDNA primer pair sequences; Table S3: Environmental variables used in the model and their contribution; Table S4: Neutrality test for the four haplogroups of *Elymus breviaristatus*; Figure S1: Pairwise haplotype mismatch distributions for populations of *Elymus breviaristatus*.
