# Supplementary figures and images for: Effects of Climatic Change on Phylogeography and Ecological Niche of the Endemic Herb Elymus breviaristatus on the Qinghai-Tibet Plateau
Source: Plants (Basel). 2023 Sep 20;12(18):3326. doi: 10.3390/plants12183326 (PMC10535585; doi:10.3390/plants12183326)

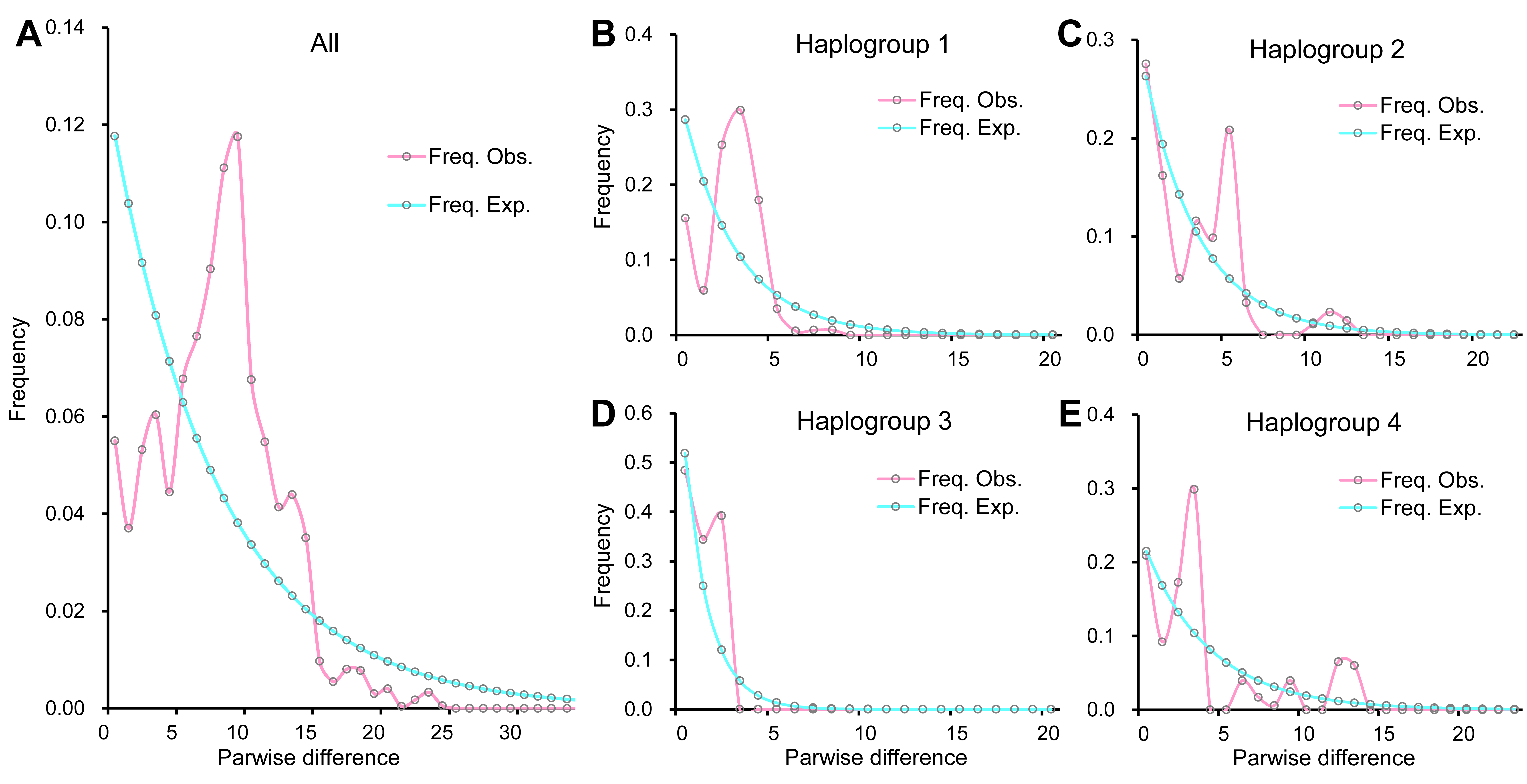

Supplement: Supplementary file 1 [file plants-12-03326-s001.zip › Figure S1 Pairwise haplotype mismatch distributions for populations of Elymus breviaristatus.png]
